# Supplementary material for: Leprosy in a prison population: A new active search strategy and a prospective clinical analysis
Source: PLoS Negl Trop Dis. 2020 Dec 10;14(12):e0008917. doi: 10.1371/journal.pntd.0008917 (PMC7771850; doi:10.1371/journal.pntd.0008917)
Supplement: Attachment — Submitted filename: Author Responses Sep 22, 2020.docx [file pntd.0008917.s001.docx]

September 22, 2020.

Carlos Franco-Paredes

Associate Editor

*PLOS Neglected Tropical Diseases*

Christine Petersen

Deputy Editor

*PLOS Neglected Tropical Diseases*

Sub: PNTD-D-20-00759

Dear Editors,

We would like to thank you for all comments about our manuscript: “Leprosy in a prison population: an active search strategy and a prospective clinical analysis” because they appointed some important aspects to improve it. A native English speaker reviewed all the paper.

I confirm that either I am the photographer of 'Figure 2A,B,C,D'.

The requested edits were considered and they are in a red in the manuscript.

**Remarks**

Reviewer's Responses to Questions

**Key Review Criteria Required for Acceptance?**

As you describe the new analyses required for acceptance, please consider the following:

**Methods**

-Are the objectives of the study clearly articulated with a clear testable hypothesis stated?

-Is the study design appropriate to address the stated objectives?

-Is the population clearly described and appropriate for the hypothesis being tested?

-Is the sample size sufficient to ensure adequate power to address the hypothesis being tested?

-Were correct statistical analysis used to support conclusions?

-Are there concerns about ethical or regulatory requirements being met?

**Reviewer #1:** There was a good objective and it gave a lot of interesting and locally needed side information. Population was well described could even be less.

I have no problems with ethical requirements. But in Manilla (World leprosy congress some had)

Author’s comments: Sorry, we did not understand your comments properly, but we evaluated all penitentiary population in that time with no ethical problem according Brazilian regulations.

**Reviewer #2:** mentioned need to be elaborated fully.

Author’s comments: Sorry, but we did not understand what the reviewer meant. About some part? All the manuscript? On the other hand, we considered your comments bellow and we rewrote the discussion and conclusion. Thanks a lot.

**Reviewer #3:**

- Are the objectives of the study clearly articulated with a clear testable hypothesis stated? Yes. However, the authors should be more clear with the writing of the objectives.

Author’s comments: Thanks. We rewrote about objectives after your comments.

- Is the study design appropriate to address the stated objectives? Yes.

- Is the population clearly described and appropriate for the hypothesis being tested? No.

- Is the sample size sufficient to ensure adequate power to address the hypothesis being tested? Yes. However the authors did not show the sample size calculi.

Author’s comments: We described the penitentiary population and that time we proposed evaluate all the prisoners.

- Were correct statistical analysis used to support conclusions? No, it was insuficient.

Author’s comments: Thank you and we decided to analyze the paper statistically after your suggestion.

- Are there concerns about ethical or regulatory requirements being met? Yes.

Author’s comments: We did not have any problems with the ethical aspects in Brazil.

--------------------

**Results**

- Does the analysis presented match the analysis plan?

- Are the results clearly and completely presented?

- Are the figures (Tables, Images) of sufficient quality for clarity?

**Reviewer #1:** As mentioned one table particularly need adaptation (table 6)

Author’s comments: Thank you very much. We made many modifications in the table 6 becoming clearer now.

**Reviewer #2:** Tables need further clarity

Author’s comments: Thanks. We reviewed the tables e made changings.

**Reviewer #3:**

- Does the analysis presented match the analysis plan? Yes.

- Are the results clearly and completely presented? No. More associations may be explored.

Author’s comments: Thanks. We understood and after your suggestions we could improve the paper.

- Are the figures (Tables, Images) of sufficient quality for clarity? No. Data and statistical test should be inserted.

Author’s comments: Sure, we decided to analyze the paper statistically after your suggestion. Thank you.

--------------------

**Conclusions**

- Are the conclusions supported by the data presented?

- Are the limitations of analysis clearly described?

- Do the authors discuss how these data can be helpful to advance our understanding of the topic under study?

- Is public health relevance addressed?

**Reviewer #1:** Conclusions are discussed and relevant

Author’s comments: Thanks.

**Reviewer #2:** Conclusion that LSQ questionnaire was useful was not substantiated.

Author’s comments: You are right. Thank you. Reading the paper again, we perceived it and we made changings in results and discussion parts as described below.

In results part: In total, 1,250 (89.3%) prisoners were evaluated in the morning, afternoon and also in the evening to clinically evaluate the individuals who work outside the unit. In the course of the action, 34 new cases were diagnosed, resulting in a new case detection rate of 2.7% within the CPP-Jardinópolis population.

There was an excellent rate of completion of the questionnaire by the inmates of this facility (89.3%), with an average number of checked responses of 2.7 questions per participant. Interestingly, among the 31 new cases of leprosy who responded, only 1.5 responses were found on average, which was slightly lower. Although none of the participants were informed that the nature of the survey was about leprosy, specific questions asked were those about signs and symptoms routinely found in leprosy patients, such as the neurologic symptoms related to the presence of tingling, numbness in the hands and/or feet, pain in the nerves, sensation of pricks and needles, and hypopigmented or red and scaly spots on the skin, responses that were found in more than 30% of questionnaires in addition to the main known symptom, “anesthetized area in the skin”.

**Reviewer #3:**

- Are the conclusions supported by the data presented? Yes, however, the conclusion is too long.

Author’s comments: Thanks. We reduced it.

- Are the limitations of analysis clearly described? No.

Author’s comments: We described about it in the last paragraph of discussion part.

- Do the authors discuss how these data can be helpful to advance our understanding of the topic under study?

- Is public health relevance addressed? Yes.

Author’s comments: Thanks.

--------------------

**Editorial and Data Presentation Modifications?**

Use this section for editorial suggestions as well as relatively minor modifications of existing data that would enhance clarity. If the only modifications needed are minor and/or editorial, you may wish to recommend “Minor Revision” or “Accept”.

**Reviewer #1:** I think it can be more to the point and more concise

Author’s comments: We rewrote some parts as described below.

**Reviewer #2:** (No Response)

**Reviewer #3:** No comments.

--------------------

**Summary and General Comments**

Use this section to provide overall comments, discuss strengths/weaknesses of the study, novelty, significance, general execution and scholarship. You may also include additional comments for the author, including concerns about dual publication, research ethics, or publication ethics. If requesting major revision, please articulate the new experiments that are needed.

**Reviewer #1:** Dear Authors,

I was very interested to read your paper since I had heard in Manilla about it. but I had difficulties reading it. Being not an English speaker I felt some of the sentences have to much Portuguese influence, making it not very clear what was actually meant. One of the authors is a native English speaker. It would be good when he has a second look at the English and for the main author I like it more concise and much less wordy. It should be shortened.

Author’s comments: Thank you for this comment. A native English speaker reviewed all the paper.

I was impressed by the sensitivity of the questionnaire and learned from the results of the questions..

Interesting was the observation that the leprosy was most likely not acquired in the prison despite the living conditions mentioned. The anti PGL-1 was high in some of the non-patients. Could it be that that there is continues contact with this antigen either in prison or since they are also allowed to go out to their home environment. Then it would be worthwhile to look at their contacts at home.

I agree that it is frightening that there are so many not diagnosed leprosy patients from Sao Paulo state.

Some remarks:

1 Title: in a prison Check the second half: epidemiological and a prospective clinical analyses (it is to much)

Author’s comments: Thank you for this comment. We changed the title for: “Leprosy in a prison population: a new active search strategy and a prospective clinical analysis”

6 would say in a prison population

Author’s comments: Ok, we accept it

8 A questionnaire

Author’s comments: Ok.

39 is imprisonment not better then reclusion?

Author’s comments: Ok

46 it is bon ton to include M. lepromatosis. For me it is not necessary but some like it.

Author’s comments: Ok

47 infectious and contagious is a repetition just another word

Author’s comments: Ok

56 there have been studies but may be not published . In other countries there are quite a number ao. In the Indian leprosy journal . Look in Pubmed.

Author’s comments: We had already cited Indian papers about it, but in this line we would like to say about Brazilian penitentiary population.

66 see above, it is in the Indian literature

Author’s comments: We have accepted this comment and added. “In the literature there are few and old leprosy epidemiological studies conducted of a prison population to determine if their risk is higher than the general population.”

110 just a remark: without smears or equivalent you may underdiagnose leprosy. You have mostly prevented this by PCR just mention this.

Author’s comments: The diagnosis of leprosy is essentially based on clinical findings as proposed by WHO. Additionally, we have used the PCR, more sensible than slit skin smear, but this positivity is still low mainly to consider early of leprosy diagnosis, which improve the risk to underdiagnosis leprosy. We also included the complete definition of leprosy case according WHO: “...and/or c) positive skin smears [7].”

117 low resistant T by some are classified by BT and could be in BP.

Author’s comments: About classification of leprosy, we decided to use only the Madrid (Congress of Madrid 1953) [9] and the Indian Association of Leprology (IAL 1982) [10] classifications.

We also changed the paragraph for: “... as follows: indeterminate (I), tuberculoid (T), borderline (B), lepromatous (L) and pure neural leprosy (PNL); and broadly classified according to WHO operational criteria [PB (I and T) and MB (B and L)] [4].”

143 was it one or both earlobes? Doing only the earlobes you may miss positivity.

Author’s comments: Thanks a lot for this comment. We just saw it was wrong because in our protocol we have collected from both earlobes and at least one elbow and lesion SSS samples.

So, we changed the test for: “Total DNA extraction of earlobes and at least one elbow and/or lesion SSS samples...”.

144 just a question the 3 patients who did not participate were they different from the other patients.

Author’s comments: Nothing different was observed among them. They just did not answer the questionnaires.

Table 1 do you have an explanation why the NLG have more positive complaint answers?

Author’s comments: The symptoms and signs described in the questionnaire are very unspecific because our goal was to screen the population about the risk of leprosy, but we also found other diseases as diabetes, HIV, hepatitis infection, carpal tunnel syndrome and others.

Table 2 just to remark you have included here also the patients who did not want to participate.

Author’s comments: Yes, we considered all individuals for clinical evaluation (n=1250), including 23 who refused blood collection.

Table 3 is this table needed? There is no significant difference between the groups can that not be just mentioned.?

Author’s comments: We agree with you (that there is not difference between the groups), but using the table presentation become easier to show many details about confinement time and number of prisoners

Table 4 My complements for the clinical testing.

Author’s comments: Thank you so much.

220 Now I noticed SSS were from 4 sites. Were they mixed together before testing?

Author’s comments: Just to increase the possibility to find M. leprae-DNA, and on the other hand, we have made only one reaction reducing reaction costs.

Table 6 In the second line I see No of patients with sign and symptoms. Are this the number of patients seen? Than you followed only 2 for a year. The last line with manifest symptoms the first column comes to 0. When I later see improvement in some patients . I think the table should be more clear. In some patients I see deterioration but later no improvement afterwards. Not seen the patient back? Does this table contribute?

The “Ichthyosis” is due to clofazimine? Or due to decreased inflammation?

Author’s comments: You are right and we have made some changings in the table to become it clearer. About zero in the first column and last line, it means that nobody related any specific signs or symptoms for leprosy in the beginning of MDT.

Yes, you can see improvement later with MDT follow-up in some but in other patients you can see some deterioration as a consequence of treatment and neural inflammation.

This table contributes to show the clinical alterations of the patients appeared after the start of MDT, mainly about neurological symptoms.

Unfortunately for us, the patients got their freedom soon before than one year and, so, we could not follow them for complete MDT.

About “Ichthyosis in islet” we think that it happens because both factors, clofazimine side-effect and also an immunological and inflammatory consequences defining the circumscribed area impaired by leprosy.

243 Why is a semi open prison to study diseases related to confinement better then a closed one?

Author’s comments: This kind of prison regime in Brazil is better because almost all these prisoners are in the end of their confinement period after a long period in closed regime

259 I see now that you mentioned some of the Indian papers.

Author’s comments: Ok

271 which doe actually highlight what. You just corroborate.

Author’s comments: Ok. We understood and have changed in the manuscript. We would like to say: "…corroborating that found in leprosy literature in general population, which highlights the importance of evaluating this type of population in confinement.”

284 Dysautonomies : other word? I understand what you meant to say.

Author’s comments: Thanks. We changed to just Dysautonomia on line 120.

286 Alizarin test needs explanations or reference. To date it is mostly used in Brazil in other programs it is not used anymore while it is a sensitive test.

Author’s comments: The alizarin test is a kind of thermoregulatory sweat testing (TST) used to evaluate the integrity of central and peripheral sympathetic sudomotor pathways from the central nervous system to the cutaneous sweat glands. The core body temperature is raised by increasing the ambient room temperature which in turn raises blood and skin temperature or after physical exercise for 10 to 20 minutes. A powder moisture of alizarin red powder, corn starch, sodium carbonate, (1:2:1) is applied covering leprosy lesion and surround before the exercise. As the result, sweating causes the indicator to change its color from yellow to dark red for alizarin red on the normal areas (surround) while on the leprosy lesion due the anhidrosis or hypohidrosis maintain the original yellow powder color.

286 With shock on palpation you mean Tinel sign?

Author’s comments: No, it means “electric shock-like pain”. We have changed it on the manuscript.

311 It was also not possible to test their contacts at home?

Author’s comments: No, because they were far coming from other towns and states.

318-326 This needs a clear understandable table 6

Author’s comments: We have changed Table 6 becoming clearer.

Please make this interesting article more to the point.

What is the difference between these authors contribute equally to this work and

& the ones contributed also equally?

Author’s comments: We have informed just according the title page of the PLOSNTD formatting guidelines.

**Reviewer #2:** An interesting paper on active leprosy case search in prison population, Providing good data with the help of questionnaire and clinical examination supported by lab tests. The fact that out about 1250 prisoners, 34 MB leprosy patients were newly diagnosed based on clinical / neurological examination with lab support is an important finding in this closed community. The paper needs to highlight and discuss this aspect more fully.

Author’s comments: Thank you very much. Sure, we made some improvement on the discussion.

**Reviewer #3:** The authors investigated, by means of Leprosy suspicion questionaire (LSQ), a hidden prevalence of leprosy in a prison population, important findings, since that, this individuals are not assessed for no one infectious disease as a routine.

Bellow are my comments by subsection:

Abstract

In line 5, the word “investigation” instead “search” may be better for this contexto of diagnosis.

Author’s comments: No, in the context of public health it is better to use “search” instead of “investigation”.

In the section Methods of the abstract, authors should state the type of study. It seems to be a cross-sectional for leprosy diagnosis by means of Leprosy suspicion questionaire (LSQ). It could be important define the range of data collection even if the authors have stated they performed a –follow-up time. The follow-up time make part of cross-sectional for investigation of cases during the declared time.

On the other hand, if authors think they carried out a prospective cohort, what I can not believe, the time zero and time-to-evento/outcome (signs and symptoms) must be declared. The proof that this study is a cross-sectional, refers to the fact the authors used the prevalence term.

Author’s comments: We agree with you and we added in the manuscript “…**Methods:** A cross-sectional study using a questionnaire…” in line 7.

Also, we added in line 102 the information about collecting time “…The active leprosy search action in CPP was planned in phases from April to May 2016…”

About follow-up of the patients we added the time also in line 250: “…All patients were followed-up by a dermatologist and a leprosy physician from May 2016 to July 2017, during the clinical and therapeutic follow-up for 12 months, with reported clinical manifestations described in Table 6…”

Introduction

From line 68 to 69 authors quoted they “evaluate the effectiveness of a new active search strategy...”. I disagree with the authors, because they evaluate a prison individuals, using a Leprosy Suspicion Questionnaire (LSQ), which help them to diagnosis and characterizing clinically and epidemiologically this sample, besides of serological and PCR investigations. Effectiveness refers to application of clinical trials or mathematical models to prove better performance using numerical data.

Author’s comments: Sorry, but we tried to measure the “effectiveness” instead of “efficacy” of the LSQ as an epidemiological screening tool to increase the chance of leprosy diagnosis.

Subjects and methods

From line 74 to 82, the section “Ethics, consent and permissions” generally is placed in the end of the Methodology. Although this section is considered important, readers would rather start the reading with the section called type of study or sample.

Author’s comments: Ok. We decided to put this section about “Ethics, consent and permissions” firstly because it is a study involving prison population.

About type of study, we added this topic. Line 87/88: “**Type of study** - This study was characterized as an observational cross-sectional comparative study…”

I suggest to authors start the section "subjects and Methods" with a subsection “ Type of study and sample” stating if cross-sectional, retrospective cohort, prospective or another type of study. Moreover, authors should state who is the sample, that is, who is the LG and NLG. The total number of patients per group may be declared if authors agree with this suggestion.

Author’s comments: As described before, we added this subsection “**Type of study**”, thank you very much. About LG and NLG in third paragraph of “**Results**” section we added more information about them: “…Individuals were grouped in two groups for comparative analysis: those diagnosed with disease - Leprosy (LG, n=34) and those without no defined criteria of leprosy - Non-Leprosy…”

The authors should write a new subsection intitled “Phases of data collection” placing the text from line 89 to 101.

Author’s comments: We changed the text about phases of data collection in line 104 including the month of start of the study and we supposed to improve it and become better about phases of the study. We also renamed the section “**Characterization of place of action**” (line 109) to “**Characterization of place and action phases**”.

About the follow-up phase we included the period in “**Follow-up of patients**” section in the first paragraph. “…All patients were followed-up by a dermatologist and a leprosy physician from May 2016 to July 2017, …”

The subsection “Diagnostic criteria for leprosy” must be maintained, except to the text from line 117 to 119 which is more interesting if inserted in the new subsection "Type of study and sample or similar".

Author’s comments: Ok. We have added it.

A subsection, “Statistical Analysis” should be created on this section “Subjects and Methods”.

Authors should drive attention in the statistics text placing better structure to express what tests were applyed in the manuscript.

For instance, The Mann-Whitney U test is used to comparing differences between two independent groups regardind to sum of ranks. New statistical tests should be explored as suggested in this revision, for example, Binomial test.

What was the statistical software used for this analysis?

Author’s comments: Ok, we created a new subsection in the end of “Subjects and methods” section. Thanks. We used the GraphPad Prism 8 software (GraphPad Software, San Diego, California) and we included this information in the manuscript.

Results

In the table 2, authors should calculate a p-value for each line in relation to the variable “Age range” with the goal to confirming that there are no diferences (or there are differences) among each age group. I recommend the use of Binomial test to give evidence that there is/or there is no difference between two percentages. The same statistical analysis should be applied to the state of birth, since that if there is difference between two percentages for each state, it will indicate a possible association encompassing NLG or LG with state of birth. In the lines where the percentage is equal zero there are no p-values. The statistical test should be pointed out in the section "statistical analysis" together Wilcoxon-Mann-Whitney test.

Author’s comments: Ok, Thanks for your comments. We tested and added information about Binomial test in “statistical” sections and also included it in Table 2.

In the table 3, if the data are non-parametric, there is no reason to show mean, but only median and maximum and minimum values. Why do the authors did not compare the groups medians related to the times exposed in table 3? It is useful indicating the statistical differences between the two groups.

Author’s comments: Thanks for your important comment about “mean” and we removed these respective lines. In the table 3, we demonstrated the time of the confinement and evaluating the data they presented an insufficient time for exposure and clinical presentation of leprosy in jail.

In table 4, It could be more intersting to making a table keeping the line variables associated with clinical criteria, however, inserting 2 columns dividing the LG into seropositives and seronegatives PGL-1 variable. This suggestion may help you find out new findings and confirm potential associations. A recommended test may be Binomial or Chi-square test. The first one is more intersting due to compare two percentages per line.

Author’s comments: Thanks for this comment. We included this analyzes in table 4.

Figure 3 – Authors should provide a self-explanatory legend figure and indicating the name of statistical test - Mann-Whitney test -, besides the "U",given by the test, to becaming clear for readers how big is the difference between the groups.

Author’s comments: Thanks for this comment.

In table 5, I suggest to the authors provide a statistical test to verify if there was difference between NLG versus LG in relation to PGL1 percentuge results. 44,12% of positivity in LG is different statistically from 19,6% from NLG ?

The binomial test may be useful.

The p-value and the Z value provided by the Binomial test should be shown at the two last columns of the table 5.

Another option would be the authors disscuss indicators as sensibility, specificity, false positive/false negative rates.

Author’s comments: Thanks for this comment.

From line 224 to 233, this information could be placed in the section “Subjects and Methods”.

Author’s comments: We wrote about in this section (lines 122 to 126), but we cited about these procedures again to become easier for the readers.

The table 6 showed a follow up time in reference to clinical data of treated patients. However, this data could be interesting whether authors had performed a follow up associated with an outcome and provided us a survival analysis. On the other hand, these informations is not important in this context.

Author’s comments: Thanks a lot for your comments.

About Table 6, according reviewer’s suggestions we have made some changings in the table to become it clearer. In leprosy it is unusual to work with a “survival analysis” because the chronicity of the disease. On the other hand, we do not have complementary exams with high sensitivity covering only around 50-60% of patients.

We find this table important because it brings us some data about the influence of MDT (only antibiotics using) on the peripheral nerve. We started with zero in the first column and last line; it means that nobody related any specific signs or symptoms for leprosy in the beginning of MDT. After, you can see improvement later with MDT follow-up in some, but in other patients you can see some deterioration as a consequence of treatment and neural inflammation.

This table contributes to show the clinical alterations of the patients appeared after the start of MDT, mainly about neurological symptoms.

Conclusions

The conclusions are too long. Authors must summarize the findings.

Author’s comments: Considering all data, we tried to put it shorter cutting at least 5 lines. Thanks.

Thank you for your consideration. I look forward to hearing from you.

Sincerely,
