# Supplementary material for: Leprosy in a prison population: A new active search strategy and a prospective clinical analysis
Source: PLoS Negl Trop Dis. 2020 Dec 10;14(12):e0008917. doi: 10.1371/journal.pntd.0008917 (PMC7771850; doi:10.1371/journal.pntd.0008917)
Supplement: Attachment — Submitted filename: Author Responses Oct 22, 2020.docx [file pntd.0008917.s002.docx]

October 16, 2020.

Carlos Franco-Paredes

Associate Editor

*PLOS Neglected Tropical Diseases*

Christine Petersen

Deputy Editor

*PLOS Neglected Tropical Diseases*

Sub: PNTD-D-20-00759

Dear Editors,

We would like to thank you for all comments about our manuscript: “Leprosy in a prison population: an active search strategy and a prospective clinical analysis” because they appointed some important aspects to improve it.

The requested edits were considered and they are in a red in the manuscript.

**Remarks**

Reviewer's Responses to Questions

**1**. We note that the following authors have not yet confirmed their authorship on this manuscript: Jaci Maria Santana. At this point this request is not urgent; we have re-sent their co-author confirmation email containing an auto-confirmation link for them to click, and we will reach out to them at a later stage if we don't hear from them. We wanted to mention this in case you are aware of them experiencing technical issues with confirming authorship via the link. If so, please do ask them to contact us directly via email and we'd be happy to assist.

Author’s comments: Jaci Maria Santana confirmed her authorship on this manuscript.

**2.** Please provide your figure file Figure 1 in .tif or .eps format only. For more information about how to convert your figure files please see our guidelines: Once you've converted your files to .tif or .eps, please also make sure that your figures meet our format requirements

https://journals.plos.org/plosntds/s/figures

https://journals.plos.org/plosntds/s/figures#loc-file-requirements

Author’s comments: We provide file Figure 1 in .tif format.

**3.** We do not publish any copyright or trademark symbols that usually accompany proprietary names, eg (R), (C), or TM (e.g. next to drug or reagent names). Therefore please remove all instances of trademark/copyright symbols throughout the text, including "GoTaq®" on page 8.

Author’s comments: We have removed it.

Thank you for your consideration. I look forward to hearing from you.

Sincerely,
